# Quantifying Extinction Probabilities from Sighting Records: Inference and Uncertainties

**DOI:** 10.1371/journal.pone.0095857

**Published:** 2014-04-30

**Authors:** Peter Caley, Simon C. Barry

**Affiliations:** 1 Commonwealth Scientific and Industrial Research Organisation Division of Computational Informatics, Canberra, Australia; 2 Commonwealth Scientific and Industrial Research Organisation Biosecurity Flagship, Brisbane, Australia; Bangor University, United Kingdom

## Abstract

Methods are needed to estimate the probability that a population is extinct, whether to underpin decisions regarding the continuation of a invasive species eradication program, or to decide whether further searches for a rare and endangered species could be warranted. Current models for inferring extinction probability based on sighting data typically assume a constant or declining sighting rate. We develop methods to analyse these models in a Bayesian framework to estimate detection and survival probabilities of a population conditional on sighting data. We note, however, that the assumption of a constant or declining sighting rate may be hard to justify, especially for incursions of invasive species with potentially positive population growth rates. We therefore explored introducing additional process complexity via density-dependent survival and detection probabilities, with population density no longer constrained to be constant or decreasing. These models were applied to sparse carcass discoveries associated with the recent incursion of the European red fox (*Vulpes vulpes*) into Tasmania, Australia. While a simple model provided apparently precise estimates of parameters and extinction probability, estimates arising from the more complex model were much more uncertain, with the sparse data unable to clearly resolve the underlying population processes. The outcome of this analysis was a much higher possibility of population persistence. We conclude that if it is safe to assume detection and survival parameters are constant, then existing models can be readily applied to sighting data to estimate extinction probability. If not, methods reliant on these simple assumptions are likely overstating their accuracy, and their use to underpin decision-making potentially fraught. Instead, researchers will need to more carefully specify priors about possible population processes.

## Introduction

The likelihood that a species has become extinct is a key question in a number of scientific and management contexts. Conservation managers are interested in whether a rare species persists. Land managers need to determine when a weed or pest has been eradicated. In all cases the extinction of the species means that associated management resources can be moved to other priorities, meaning there is significant value in being able to infer the time of extinction.

The value of this knowledge means that a number of authors have considered this problem. Early models to estimate the likelihood of a rare species being present (e.g. [1]) explored the issues of sampling and power calculations assuming that the probability of detecting the presence of a population was known. Subsequently [2]–[4] considered weights of evidence for, and tests of the null hypothesis, that a species remained extant assuming a stable sighting probability, and extended this to a declining sighting rate arising from a declining population. The application focus of these models has been to rarely sighted species teetering on the brink of extinction with low demographic vigor (e.g. Carribean monk seals 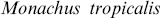 Gray 1850, black-footed ferrets 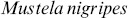 Audubon & Bachman, 1851). More recently there has been a series of papers considering optimal decision making during eradication attempts of invasive species [5]–[7]. These papers consider the question of when to cease an eradication attempt and assume that the sighting and extinction probabilities are either known or can be elicited from experts.

Review of this literature reveals a number of gaps in current knowledge. First, there has not been been a systematic consideration of using the sighting data to infer parameters of the underlying models. The existing approaches either assume that the underlying parameters are either known or can be elicited [5], or consider uncertainty in the sighting parameters implicitly in the construction of the likelihood needed to test the null hypothesis of species persistence [2]–[4], or optimize decision making [7].

Second there has been limited discussion in the literature of the plausibility of the assumed models and how this may impact on their performance (though see [8] and [9]). Methods developed in one context, such as a declining species, are being applied more broadly. This means that the underlying population processes are potentially more diverse and this may need to be reflected in the model. In contrast, the existing models in the literature are simple, presumably for parsimony and analytical tractability. As an example, [5] assume a constant probability of detection when many extinction processes would involve a declining detection rate. Likewise, recent approaches to incorporating variable sighting reliability [10], [11] assume a constant sighting rate prior to extinction.

The approach to model formulation and analysis has also been somewhat adhoc. Simple models are incrementally modified to correct identified deficiencies. For example model elaborations such as declining sighting rate ([3],[7]) are a step in addressing the issue of model plausibility but may not be the complete solution. In another example the work by [7] following [2] makes strong assumptions about priors which will not be applicable in general, and the observation model they consider is not equivalent to the model proposed by [5] due to a different formulation of the extinction process.

An example of these issues is the use of existing techniques to analyse data on invasive species. These species have potentially robust demographics that can lead to rapid changes in abundance that may or may not overlap with sighting mechanisms. Their detectability can vary in complex ways as their distributions change, and establishment may occur after a prolonged lag-phase where sightings may be few [12]. In short, populations of invasive species may be increasing despite populations being small and observations sparse, so model assumptions need be applicable to such possible scenarios. Indeed, attempts at eradication of invasive species often fail, whether they be vertebrates [other than those undertaken on islands] [13], plants [14], insects [15] or pathogens.

Developing techniques to make formal inference from sighting data can form the basis for addressing aspects of these knowledge gaps. Making inference requires specifying prior beliefs about population processes. They will provide a framework to understand how we learn from sighting data, as well as providing the opportunity to assess model fit and plausibility. The issue of fit and plausibility is key here. It is unlikely that the data alone will be able to fully resolve competing alternative models of the underlying population dynamics and observational (sighting) processes. Different processes with substantially different extinctions outcomes can plausibly produce similar sighting data in some cases. Thus we argue that the fundamental issue in analysing eradication data is the inherent uncertainty in the underlying phenomena and its impact on detectability and extinction probability over time.

This paper explores this issue. We utilize a Bayesian formulation that can accommodate some of the uncertainty seen in real world problems through the specification of prior distributions. The approach is natural in this context, and has been recommended by [4] and [16], and used in the original contribution of [2]. The advantage of the formulation goes beyond the specification of priors. The sighting data will often be sparse and provide weak information about the underlying process, leading to ridges in the likelihood where different potential processes cannot be resolved on the basis of the data. The Bayesian analysis can naturally accommodate this whereas difficulties arise if trying to use maximum likelihood techniques when there are multiple maxima or ridges in the likelihood. Examples of the issue are discussed in [17] who consider issues in analysing ring recovery data in birds and [18] who have recently illustrated the difficulties in fitting occupancy models when data are sparse and underlying models of detection are unknown.

The paper is arranged as follows. First, it considers the simple case assuming constant yearly probabilities of survival and detection, with the implicit assumption that both population size and sighting effort are effectively constant. These assumptions underpin the current default models of inferring extinction probability and underpinning decision making when a declining population cannot be safely assumed. Second, these assumptions are relaxed, and probabilities are allowed to vary as simple functions of population size which is no longer assumed to be constant or declining. We apply the two models to make inference on the fate of the recent incursion of the European red fox (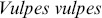 Linnaeus 1758) into Tasmania [19], an island state of Australia. We compare and contrast the results from these two sets of modelling assumptions, and discuss the justification for choosing one over the other. For completeness we also present the original approach to inferring species extinction of [2] and the subsequent hypothesis testing approach of [4].

## Materials & Methods

### Model specification—constant population

#### General

We begin by considering inference in the simple model outlined by [5]. This model assumes constant detection and population persistence probabilities prior to extinction (if this occurs). Formally, assume we have a population in a defined region and that we observe the process at certain time intervals. At each time point 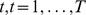 we observe whether the population was detected in that time interval, denoted by 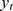. We define 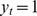 if the population is observed and 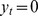 if it is not observed. We assume that during a time interval the population process under study may cease (i.e. become extinct) with probability 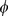 and that the probability that it is observed is 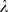 if it is extant and 0 otherwise. We assume that the species is extant at time 0 although this could be relaxed. The observed data is 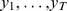. For computational convenience we augment the formulation with an additional variable 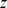 which is the time to the population becoming extinct following the most recent non-zero observation, or 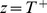 if the population is not extinct by time 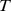. This augmentation is convenient as we will want to make inference on the value of 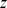 and it simplifies the algebra and sampler design. We adopt a Bayesian approach to inference and therefore need to determine the likelihood of the data to calculate the posterior distribution. The joint distribution of 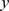 and 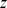 is
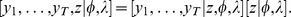



Now, further conditioning on 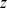 gives
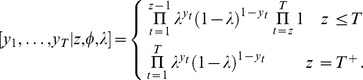
Note that we assume that if the population goes extinct during a year it is not available to be detected. If we collapse 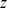 values greater than 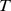 into a single category
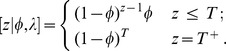



The posterior distribution of interest is therefore
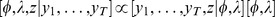
with 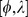 the joint prior for 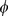 and 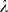. To perform inference on the parameters we construct a Gibbs sampler. Define the time since the start of the observation process to the last non zero observation as 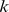. Given this 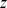 can take the values 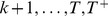. The conditional distribution 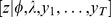 is therefore a multinomial with 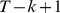 categories and probabilities
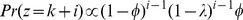
for 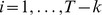 and for 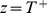:
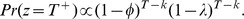
The conditional distribution of 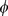 is
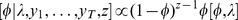
with 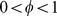. The conditional distribution of 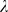 is
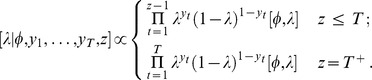
with 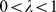.

The final issue to complete is the posterior distribution for the time of extinction conditional on the data. We define the indicator variable 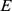 to be 0 if the species is extinct in the time period of the observed data and 1 if it is still extant. In this case
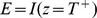
where 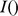 is the indicator function which takes the value 1 if the argument in the parentheses is true, and zero otherwise. The time of extinction is 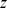 for 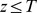. When 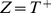 it means that extinction occurs at some time in the future. Under the model the predictive distribution of the number of years 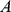 beyond 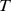 that extinction occurs is
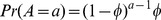
with 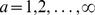.

#### Model fitting

**Figure 1:**
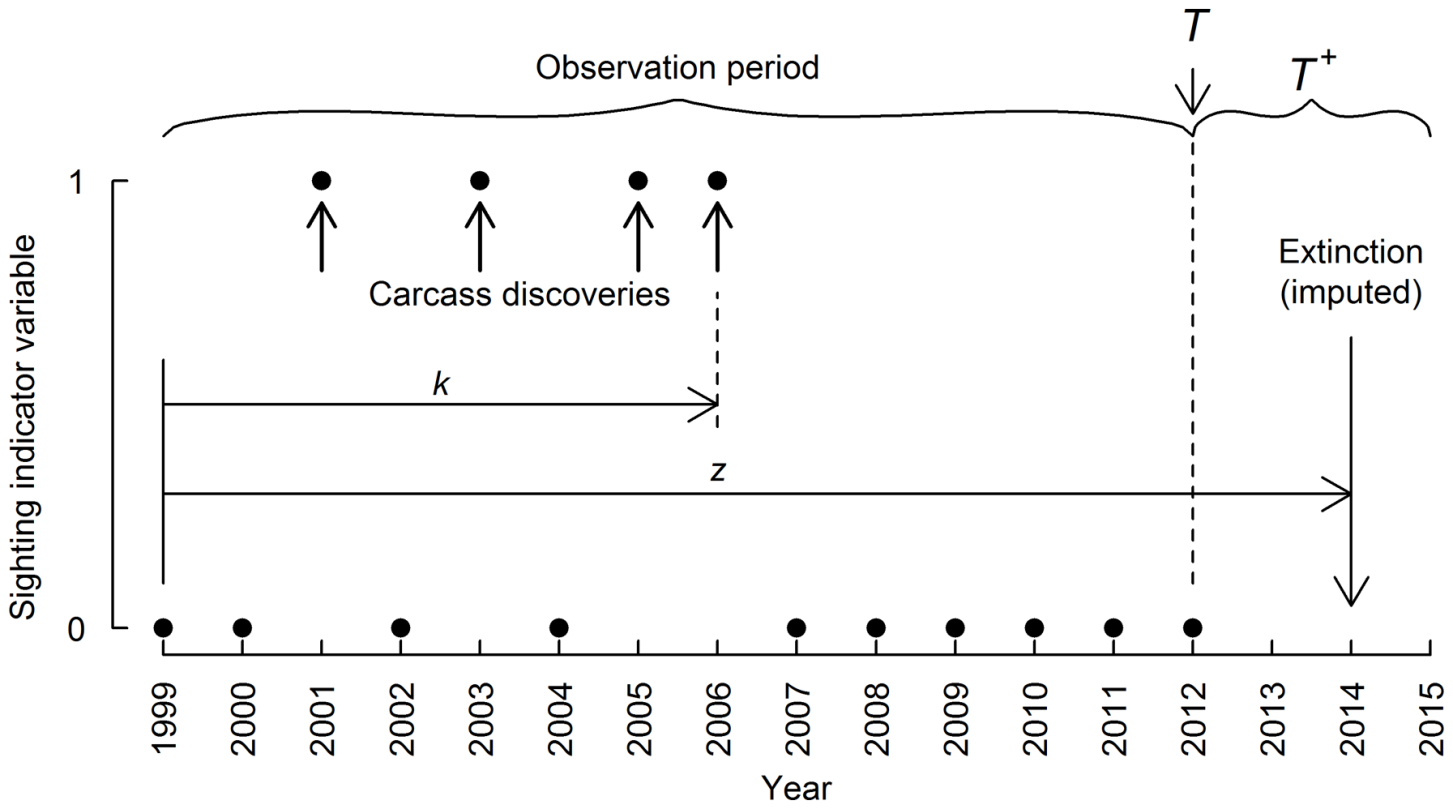
Schematic diagram of the model process and parameters. Solid circles are observed data on the discovery of red fox carcasses in Tasmania used in the worked example.

A pictorial explanation of the Gibbs sampling process is illustrated in Figure 1 using sighting data from the Case Study (below). Calculation of the posterior distributions requires specification of prior distributions for 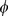 and 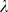. Given their range it is natural to consider a Beta distribution as priors. That is, 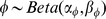 and 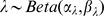. The Beta distribution is a flexible distribution and conjugate with the conditional distributions for the parameters which simplifies the sampling. The sampling is straight forward and standard methods can be used to summarise the key components of the posterior distributions. In particular the posterior probability that the species is extant is consistently estimated by averaging the indicator variable 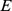. That is
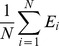
where the sum is over the 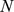 samples from the posterior distribution after a suitable burn-in period.

### Model specification—non-constant population

#### General

The simple model presented in the previous section makes a number of assumptions that may not be true in practice. In particular the assumption of a constant detection and extinction probability would often be questionable in many circumstances.

While it may be argued that the simple model provides a reasonable approximation in some cases, this is an unsatisfying approach in the absence of supporting information and analysis. A principled approach to this issue involves constructing a broader model space that spans a more plausible set of possible processes. Inference made over this model space will then factor in a more plausible range of possibilities.

As an example, [3] assumed that sightings may come from a non-stationary Poisson process with declining rate function up until the time of extinction 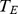:
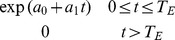
(1)where 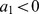. This is consistent with the sightings being distributed as Poisson where the rate parameter is proportional to the population size 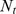 which changes exponentially:
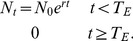
(2)In the model of [3], the exponential rate of increase (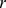) is less than zero (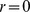 is equivalent to zero population growth). We have argued, however, that this is probably too restrictive in cases of attempts to eradicate invasive species that could quite possibly have positive rates of increase.

The assumption of exponential population growth/decline is still very strong, though it is the simplest and most tractable model to start with when relaxing the assumption of a constant population. It is, however, a more plausible model and hence requires a smaller ‘leap of faith’ (after [20]) than the first model that is most easily interpreted as assuming a constant population size. Increasing populations are often well described by stochastic exponential growth [21], as are declining populations [22]. Here, by necessity, we ignore the stochastic component, though return to its importance in the discussion. For our illustrative purposes we choose the simplest detection and survival model possible given the changing population. Let 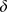 be the yearly rate of detection per unit of population, hence the now time-dependent yearly detection probability (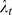) becomes:
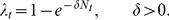
(3)


We note that 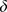 and 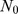 are not uniquely identifiable from sighting data alone. This is not of concern, as we are primarily interested in the ability of the model to infer the timing of extinction arising from estimates of 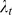 and 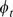.

In a similar vein, let the time-dependent yearly probability of extinction (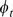) be related to the logarithm of population size on the logit scale:
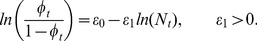
(4)


This model allows considerable flexibility (non-linearity) in how extinction probability may vary with population size. Taking the logarithm of 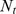 ensures that extinction becomes certain as the population approaches zero.

As in the first model we need to derive the posterior distribution. Noting that we again introduce the variable 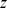 to denote the time of extinction. We have
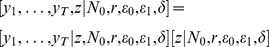
In this case
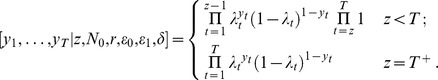
and
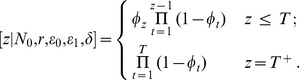
The posterior distribution of interest is:
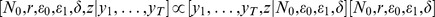
with 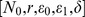 being the joint prior for the underlying parameters. To perform inference we again construct an appropriate Markov chain and sample from it.

Note that the simple model is embedded in this more complicated model. Consider we have a simple model with parameters 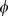 and 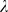. We look to find a parameterisation of the non-constant population model equivalent to this. Note that if we have 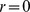, then 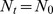 (i.e. the population is constant). If we then solve the equations
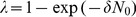
(5)and
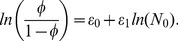
(6)for 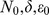, and 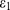 we will have an equivalent formulation. Note we have two equations in four unknowns and there are multiple solutions corresponding to a subspace of the complete parameter space in the non-constant population model when the rate of population increase is non-zero.

#### Sampler

The MCMC sampler was run as before, though with a Metropolis-Hastings step rather than a Gibbs sample as the posterior distribution of the detection and survival parameters conditional on the imputed extinction time no longer had a standard form.

### Worked example—discovery of red fox carcasses in Tasmania

#### General background

The apparent incursion of the European red fox into Tasmania has caused considerable alarm due to predicted severe negative impacts on biodiversity. Indeed, Tasmania is home to several small mammal species that are extinct on the Australian mainland other than within predator-free exclosures, with the introduced red fox inferred to be one of the main drivers of extinctions of these mainland populations [23], [24]. The evidence for the incursion is varied, including sightings, footprints, carcasses, and DNA extracted from scats etc [19], [25]. Some of the data are contentious [26]. An early scientific overview concluded that “unknown number of foxes have been deliberately and/or accidentally introduced to Tasmania since 1998 and that some of these and possibly their progeny are still living in the wild in Tasmania” [27]. This resulted in an eradication program being instigated. There is practical interest in knowing the probability that eradication has been achieved, and theoretical interest in methods of estimation. Here, we are not interested in debating the credibility of the broader evidence, but rather in exploring the information available from the simple sighting records. In particular, we choose to analyse the irrefutable evidence that fox carcasses have indeed been found in Tasmania—it were these data that alerted the authorities to the possibility that an incursion was underway.

#### Carcass discovery data

Data on the discovery of fox carcasses are taken from publicly available data provided by the Fox Eradication Branch of the Tasmanian Department of Primary Industries, Parks, Water and Environment (http://www.dpiw.tas.gov.au/). The dates of carcass discoveries span the years 2001 to 2006 (Table 1). We did not include the “Longford” fox carcass in calculations (reported as shot in July 2001 with the purported skin was produced as evidence, but not the carcass) as its authenticity has been questioned. If included the effect would have been to slightly increase the estimated detection probability, and hence increase the probability that the process has ceased given the sighting data. The year of first introduction is rumored to be 2001, although “accumulated evidence also indicates that such an act may have also occurred in 1999 and 2000” (Saunders *et al.* 2006). For our modelling purposes, the sighting data are 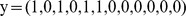, assuming the incursion started in 2001 with the first detection also in 2001 and the last year with no detection being 2012. If we assume the population incursion and associated carcass generation process started in 1999 (two years prior to the first detection), then the sighting data are 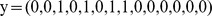. We undertake calculations using both starting times to illustrate the effect of such uncertainties on model inference (see below). This is clearly a very small dataset (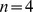 sighting years with at least 8 non-sighting years). It is, however, equivalent to the Caribbean monk seal data (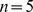) used by [2], [4], as being an invasion arising from an introduction, we have chosen the start of the observation period based on available knowledge. Such small and challenging datasets will often arise when dealing with eradicaton programs in reponse to incursions of invasive species.

**Table 1:** Details of fox carcasses found in Tasmania, including the cumulative number (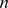), year and month of discovery, time from discovery of first carcass (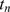), and the location of the finding.

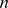	Year	Month	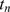	Location and name (if applicable)
1	2001	September	0	Symons Plains—the “Bosworth fox”
2	2003	October	25	Burnie—“Burnie road kill”
3	2005*	December*	51	Lillico Beach, Devonport
4	2006	August	59	Cleveland

*Although officially reported as February 2006, this carcass was first sighted in December 2005.

#### Model fitting—constant population

Given the uncertainty and contentious nature of the processes underlying generation and discovery of red fox carcasses in Tasmania, it may seem appropriate to use uninformative priors for both survival and detection (e.g. 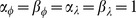, corresponding to Uniform distributions on the interval [0,1]). It can be argued, however, that 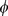 isn't very close to 1 (i.e., it is likely that the population is extant for more than one year). In a similar vein, a prior on 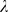 that downweights values near 0 and 1 makes sense, as if 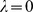 there would be no sightings and if 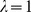 there would be annual sightings. Hence weakly informative priors seem a better approach, and we chose 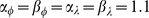, which is reasonably uninformative (flat) other than at 0 and 1, which have probability zero. Other priors may be used if further information of a credible nature becomes available. The choice of priors corresponded to an implicit prior probability of an extant population at the end of the observation period of 1.8%

We ran the sampler for 1 million iterations. Posterior distributions of detection probability, survival probability, and time to extinction were taken directly from the chains without any thinning. We also used the model to explore how the estimated probability of extinction may change in future up until 2015, assuming no further carcasses are discovered.

#### Model fitting—non-constant population

We chose largely flat priors for all parameters, largely unconstrained other than to reflect biological limits. The prior distribution for the population growth rate (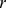) was uniform on [−1.6,0.69]—that is, the underlying trend in population growth rate lies somewhere between a 5-fold decrease and 2-fold increase each year. Without strong prior knowledge on either 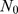 or 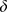 it makes sense to simply fix the initial population to one, such that all subsequent population sizes are scaled relative to this. The corresponding rate of detection 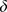 was constrained to be positive and uniform on [0.01,4.6], which corresponds to a very wide range of possible yearly population detection probabilities, with the limits corresponding to the initial population being detected with probability between 1% and 99%. A Uniform [−20,20] prior distribution was chosen for 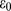 and Uniform [0,20] for 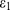. These lower and upper bounds are arbitrary and naive—we do not have well informed prior beliefs on how non-linear the probability of extinction could possibly be.

The chosen priors correspond to a implicit prior probability of 3.9% and 3.5% that the population is extant as of 2013 for a 2001 and 1999 start, respectively. The sampler was run for 1 million iterations, as the chain mixed quite slowly (this could potentially be rectified technically but is not the focus of this paper), after a “burn-in” period or 100,000 iterations. Again, following [28] we didn't thin chains.

#### Model fitting—earlier approaches

The methods of [4] can be used to first undertake a null hypothesis test of whether the population is extant and second estimate the time to extinction conditional on rejecting the null hypothesis. We also revisit the Bayesian formulation of [2], that underpins the calculations of [7], and requires a prior on the probability that the species is extant. The methods assume the sighting process to be Poisson with a constant underlying rate of carcass discovery up until extinction, if this occurs. We did not consider the method of [3] for inferring extinction under the assumption that the population (and hence sighting rate) is declining, as in the case of the red fox incursion in Tasmania, and typically in other situations of trying to eradicate an invasive species, the assumption of a declining population is inappropriate (see Introduction). Rather than discarding the first sighting in 2001, and choosing this as the start of the process, we include the first sighting and specify the start time separately, as we have reasonably strong independent information as to when this occurred. This also highlights differences in using the models of [4] to infer the extinction of a long-established species on the verge of extinction, in which case a 2001 start time would be used in our worked example, with an introduced species where the introduction date may be known with varying degrees of certainty, and possibly prior to the first sighting. For the method of [2] we explore the effect of varying the prior belief of population persistence (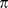) from 0.1 through to 0.9.

All analyses were undertaken using the R software [29] including use of the MASS library [30]. R code to run both samplers is provided (see Supporting Information).

## Results

### Constant population model

**Figure 2:**
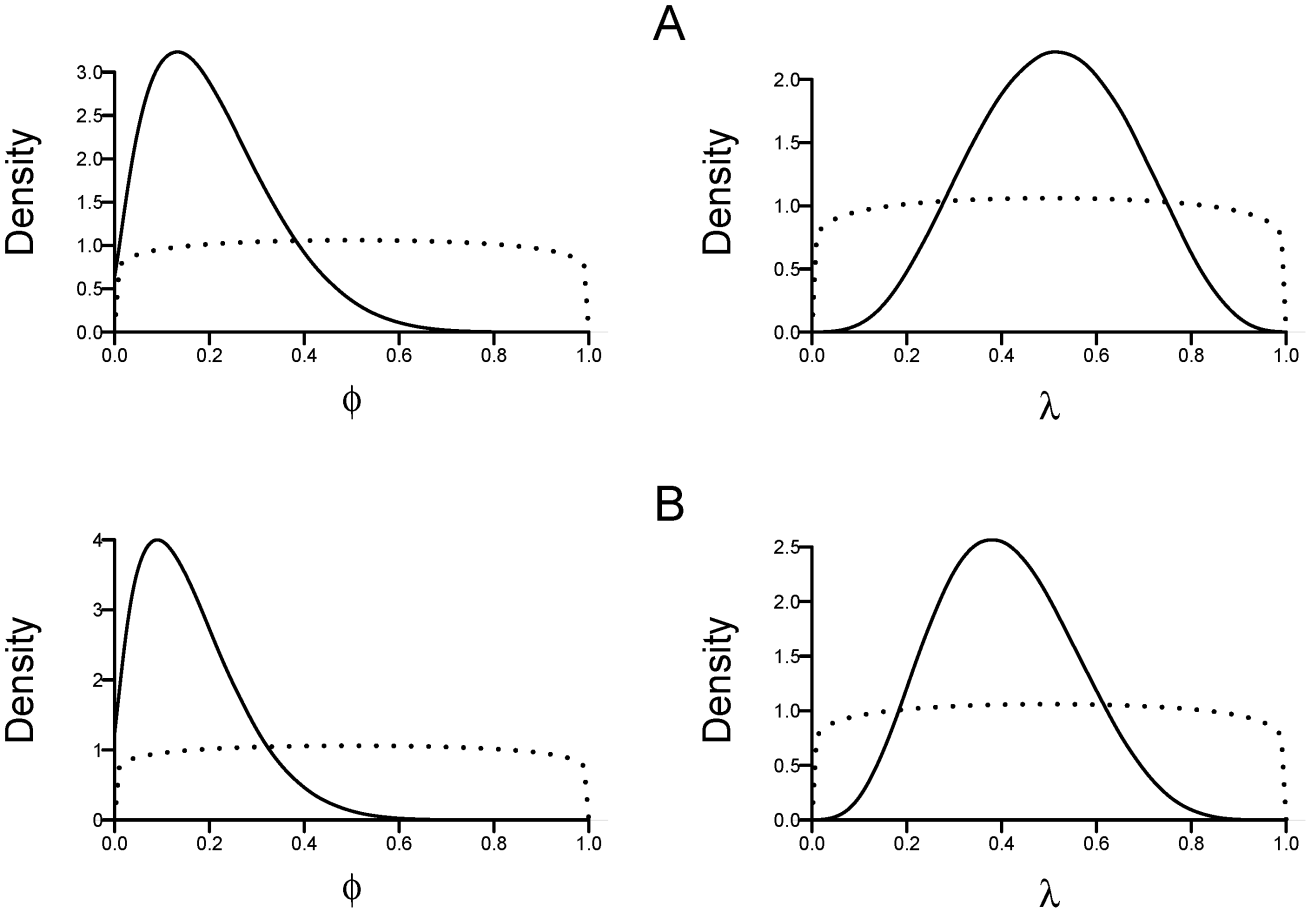
Posterior distributions for ‘constant’ population model with weakly informative priors for the yearly probability of process cessation(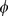) and yearly detection probability (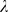). The carcass generation process is assumed to have started in either (A) 2001, or (B) 1999. Dotted lines are prior distributions.

Conditional on the model the data clearly informs the posterior distributions for 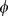 and 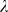 (Figure 2). Assuming the carcass generation process started in 2001 and after no detections during 2007–2012, the median yearly survival probability for the population is 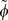 = 18.6% (95% C.I. 2.3–50.7) and the median yearly detection probability is 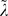 = 51.3% (95% C.I. 20.3–81.9) (Figure 2A). If the incursion started in 1999, the estimates are 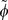 = 14.0% (95% C.I. 1.3–42.0) and 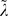 = 40.1% (95% C.I. 15.3–70.4) (Figure 2B).

**Figure 3:**
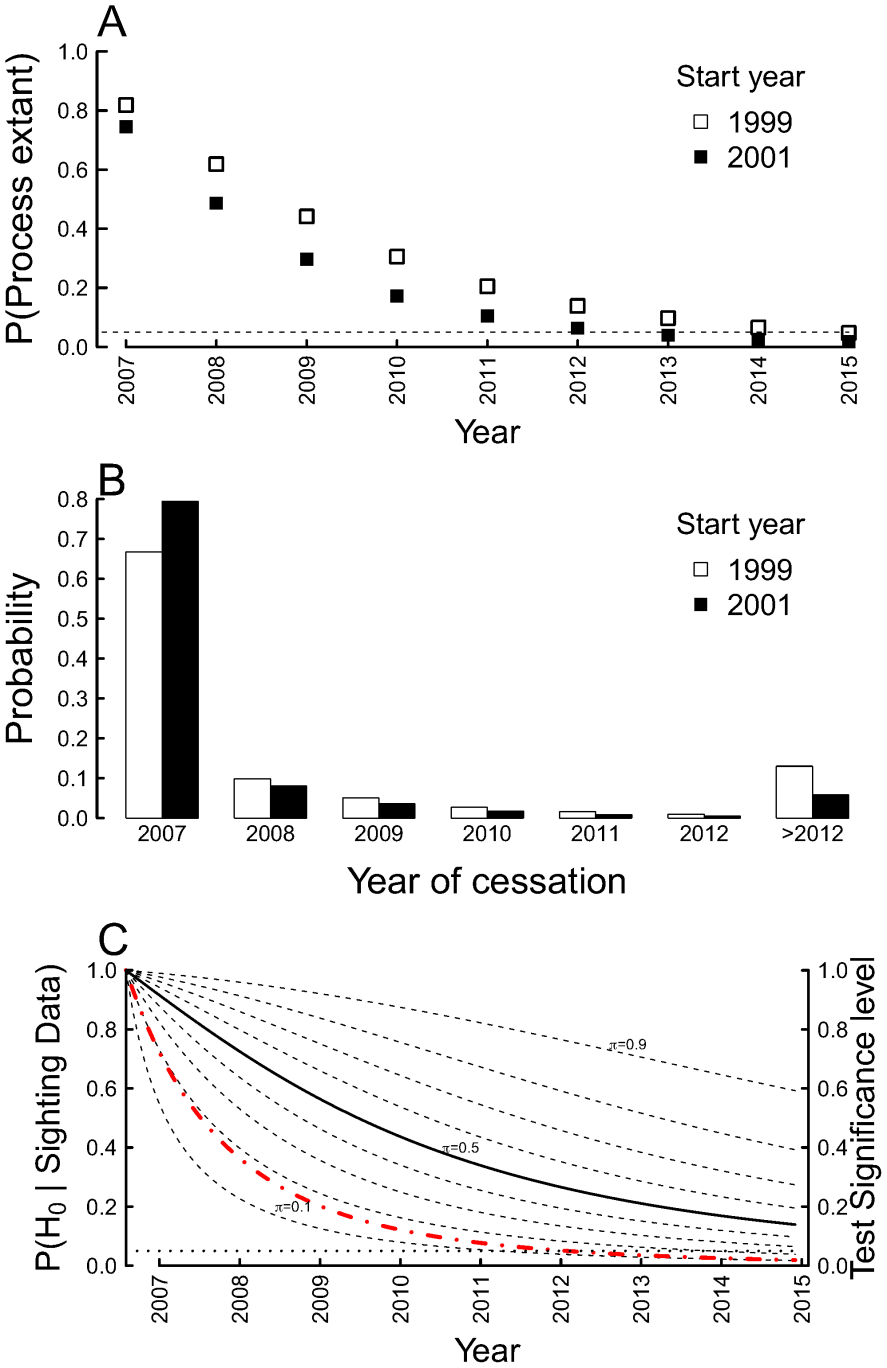
Results of simple models estimating extinction probability and timing. (A) Probability that the process generating the discovery of fox carcasses in Tasmania is extant up until 2015 (assuming no further carcasses are found) based on ‘constant’ population model with weakly informative priors. The process generating fox carcasses is assumed to start in either 1999 (circles) or 2001 (squares). The horizontal dotted line is at 5%. (B) Year of cessation of carcass generation process as of year-end 2012 assuming ‘constant’ population model, with the process first started in either 2001 (solid bars), or 1999 (black bars). Priors for yearly detection probability (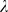) and probability of carcass generation process cessation (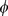) are assumed weakly informative. (C) Left vertical axis: Probability of the null hypothesis (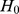) given the sighting data for different prior beliefs (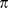) that the population is extant, for values from 0.1–0.0 in intervals of 0.1. Right vertical axis and red dashed line: Significance level for testing the null hypothesis that the process generating fox carcasses in Tasmania is extant versus the alternative that the process is no longer operating.

The probability that the population is extant as of 2013 is either 6.2% or 13.9% assuming a 2001 or 1999 start to the incursion, respectively. Should there be no further carcass discoveries, this probability will continue to drop in an exponential manner, and in 2015 reach either 1.6% (2001 start) or 4.7% (1999 start)(Figure 3A).

The most probable year the carcass generation process ceased to operate is 2007; either with probability 79.5% assuming a 2001 start or 67% assuming a 1999 start (Figure 3B).

### Non-constant population model

**Figure 4:**
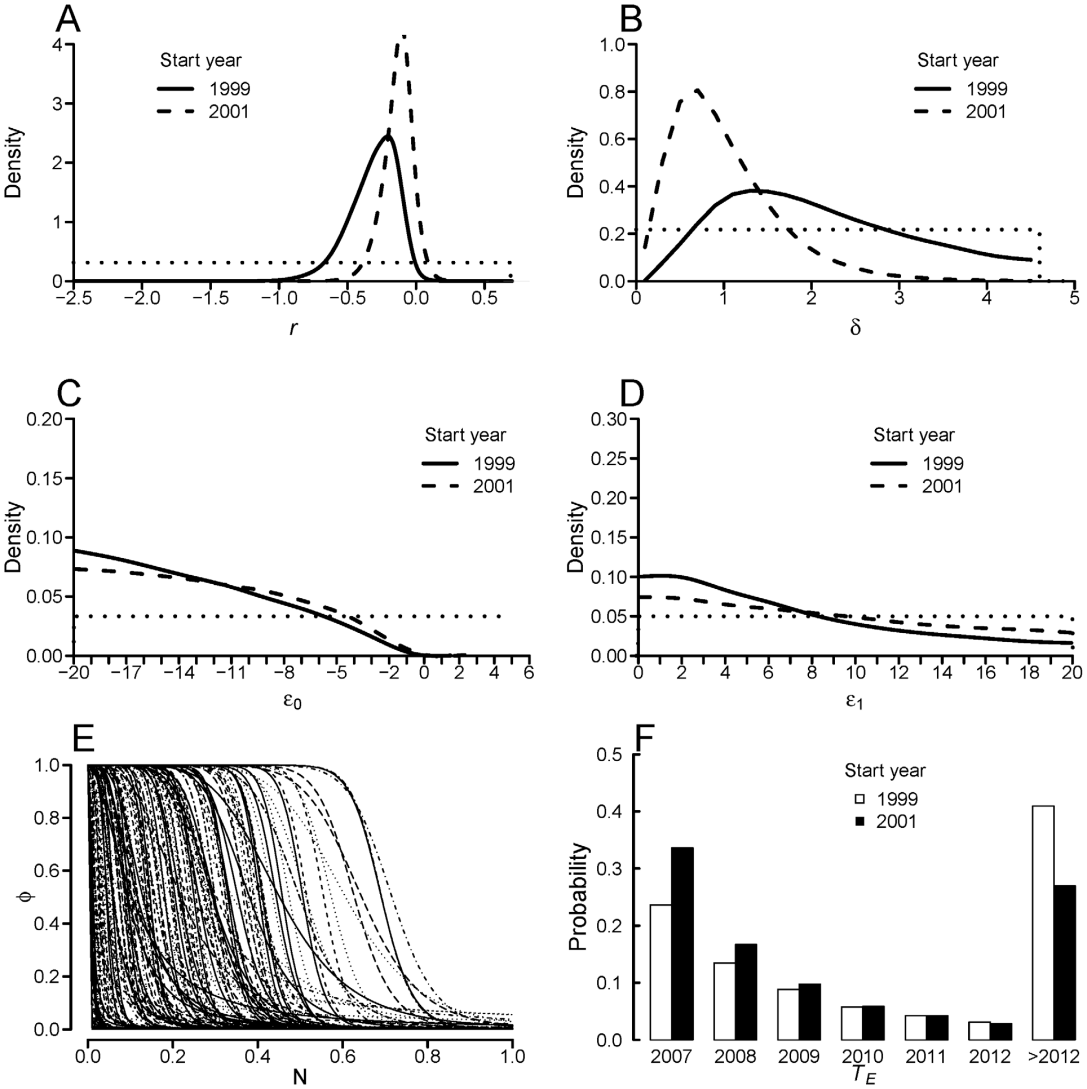
Marginal posterior distributions from ‘non-constant’ population model with weakly informative priors assuming 2001 introduction (solid line), 1999 introduction (dashed) line. Distributions are for (A) instantaneous rate of population increase (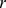), (B) detection rate (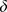) and (C)–(D) parameters (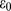, 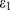) generating yearly extinction probability from population size, (E) sub-sample (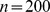 for clarity) of relationship between yearly extinction probability and population size (2001 introduction only), and (F) year of extinction (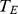). Dotted lines are prior distributions.

Allowing the population to vary prior to extinction changes our inference substantially, and the following pertain to the incursion starting in 2001. The data appear to have informed the prior for the rate of increase, which infers the population is in decline (i.e. 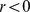), although the rate of decline remains uncertain (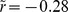, 95% C.I. −0.72–−0.04)(Figure 4A). The detection rate parameter is somewhat informed (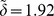, 95% C.I. 0.48–4.3)(Figure 4B). The data, however, reveal little about the shape of the relationship between population size and extinction probability, as evidenced by weakly informed marginal posteriors for 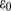 (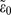 = −13.7, 95% C.I. −19.7–−3.3) and 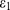 (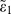 = 5.5, 95% C.I. 0.3–18.5)(Figure 4C&D). Though constrained within the bounds of the priors, the inferred possible relationships between extinction probability and population size are diverse (Figure 4E). The probability that the population is extant as of 2013 is 26.9% (Figure 4F). Assuming an earlier incursion start lessens the inferred rate of population decline (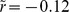, 95% C.I. −0.37–0.05)(Figure 4A), decreases the detection rate (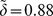, 95% C.I. 0.20–2.57)(Figure 4B) and increases the probability the population persists into 2013 to 40.7%.

**Figure 5:**
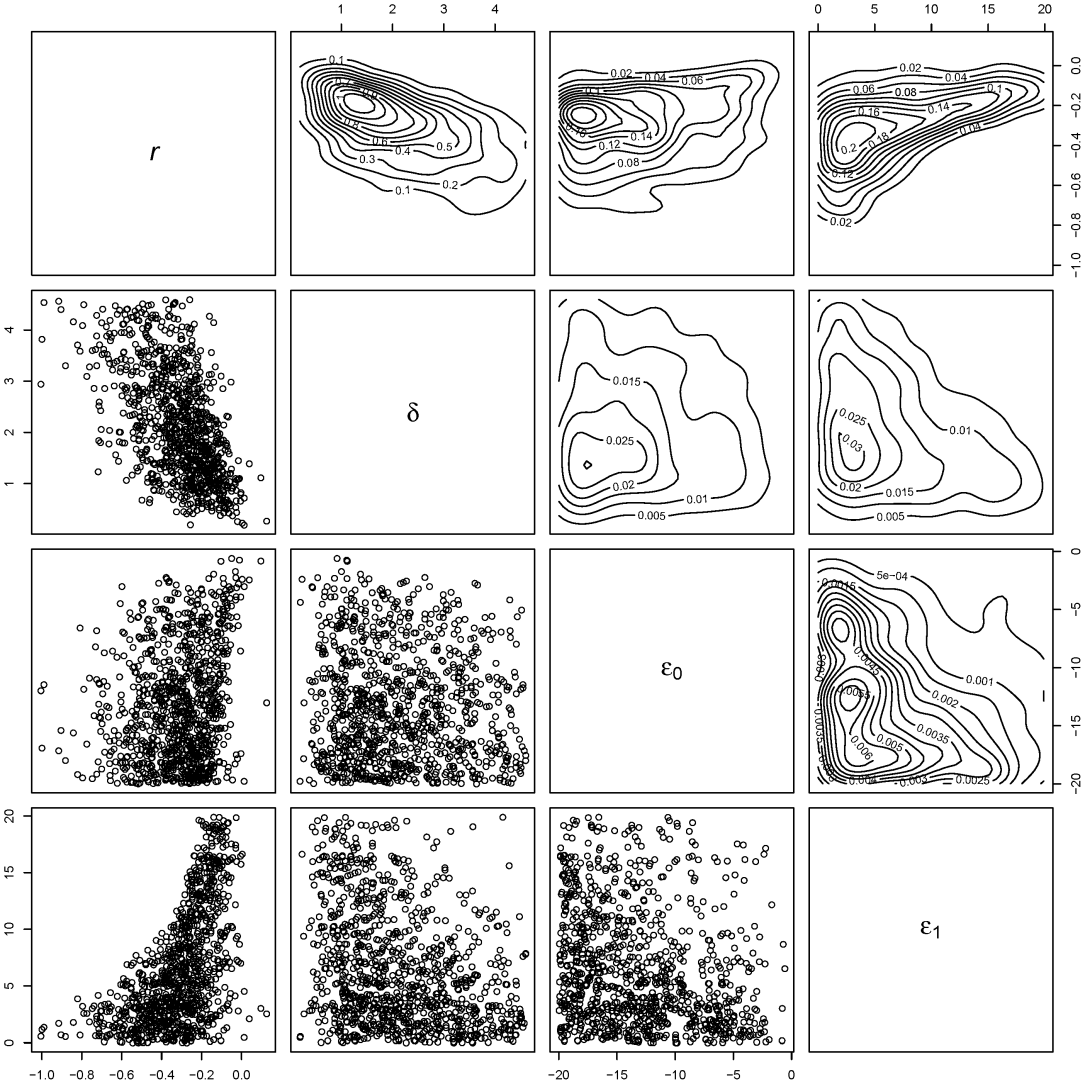
Pairwise plots of parameter values from posterior distribution for model with non-constant population and fox introduction during 2001. The lower-left diagonal panes show pairwise scatter plots from a sub-sample (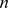 = 1000) of the posterior distribution for pairs of variables as labeled on the diagonal (thinned for visual clarity). The upper-right diagonal panes show corresponding joint posterior density surfaces.

The uncertainty in the density-dependence of the extinction probability is further illustrated by the pairwise scatter plot and associated flat joint posterior densities, remembering these estimates are on a logit scale (Figure 5). Ridges are also evident in many of the joint posterior distributions, particularly those involving the detection probability (Figure 5), illustrating the understandable difficulty the sparse data has in resolving the parameter values through the likelihood (in combination with prior information) [31]. It stands to reason that if the model has not been able to identify, and hence estimate the yearly extinction probability through the parameters 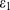 and 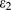, then the estimates of time to extinction are probably poorly resolved also.

### Earlier approaches

The estimated probability of the null hypothesis (that the population is extant) given the sighting data, as a function of year and prior belief of extinction (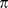), is shown in Figure 3C. The results are sensitive to the value of 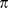 chosen, which is independent of the sighting data. Assuming 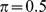 (the “Principle of indifference”), then as of the end of 2012 the estimated probability the population is extant is about 0.3. There are, however, some limited data to inform our beliefs. [32] lists two of three introductions of the red fox to islands as being successful, hence a prior for 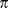 in the order of 0.5–0.8 appears reasonable, which corresponds to a probability of persistence of 0.3–0.6 (Figure 3c)—the limited extent to which the data have informed the prior illustrating the weakness in the data. The frequentist approach provides contrasting inference. As of the end of 2012, the p-value for testing the null hypothesis that the fox population is extant has fallen below 0.05—the null hypothesis is struggling to retain credibility in the face of a recent lack of fox carcasses (Figure 3C red line). If we assume the process of carcass generation has ceased, then using the parametric estimator of [4], the MLE estimate for the time this happened is 15 months (December 2007) after the last carcass discovery. The upper 95% confidence interval is 66 months (February 2012) after the last carcass discovery.

## Discussion & Conclusions

The analysis raises questions about current approaches to analysing and understanding sighting data. There is an apparent paradox between the simpler constant population model that reveals reasonably strong identification (i.e. certainty) of parameter values and inference about extinction times and the more complicated non-constant population model where there is considerably more uncertainty about the process involved and therefore the trajectory to extinction. This result can be explained by noting that the non-constant population model's parameter space accepts a wider range of possibilities about the potential dynamics of the population. The data, through the likelihood, cannot fully resolve these possibilities. As these possibilities imply a wide range of extinction outcomes, these are reflected in the posterior estimates. The more precise estimates from the simpler model reflects that it is equivalent to using a prior in the non-constant population model that effectively disallows (i.e. sets prior probability to zero) parameter sets that are not solutions to Equation 5 and 6—a considerable restriction on the form the model may take.

The implicit assumption that all other processes have zero plausibility is quite stringent and represents strong knowledge of the system in question. This would appear to be at odds with the inherent uncertainties in either rare populations with weak demographics on the verge of extinction, or invasive populations with potentially high population growth rates. It is a salient point that the simple model chosen is only one of a large number of plausible models that could lead to materially different outcomes—why the simple model is a natural starting point is not considered in the literature.

The restricted nature of the simpler constant population model would not be an issue if the inferences were equivalent between it and the more elaborate non-constant population model. This would occur if the simpler model represented a rigorous marginalisation of the more elaborate model. That is, the simpler model arises from integrating the more complex model over the parameter space of the variables being omitted. This has not occurred in this example and would appear unlikely in many cases. An alternative argument may be that the simpler model provides a reasonable approximation to the more complicated model. This would not appear to be the case in this example and there has been no discussion of this in the relevant literature to this point.

The non-constant model has a number of issues. Specification of priors is difficult and the sparse data are not particularly informative. There are additional challenges. The more elaborate non-constant population model still pays scant regard to population processes. For example, it takes no account of the possible spatial dimension of the underlying population nor stochasticity. Typically an invading population could have temporally varying overlap with sighting mechanisms, invalidating our assumption of constant sightability for a given population size. This has the ability to bias our estimates of extinction probability upwards in the case of the population subsequently establishing in areas of lower detection probability—inaccurate delineation of the infestation area is a common problem underlying failed eradication programmes. In our case study, the inference is quite sensitive to the assumed timing of the start of the sighting process. Back-dating the incursion by two years from the first sighting increases the probability that the population is extant in 2012 by a factor of around two.

Although we consider the Bayesian analysis of the constant population model useful in some circumstances, the assumptions are difficult to justify. The alternate inference from the more elaborate model is also problematic due to the sparse data and the challenges in specifying plausible population and sighting processes and associated priors. The question regarding the best way to analyse sighting data to infer extinction timing is unresolved. This paper has demonstrated the limitations of the simple models, but developing appropriate models and priors may well be case specific and require considerable resources. Thus we are not advocating indiscriminate use of the non-constant model, but rather see its value as illustrating the difficulties inherent in this field, and as a tool for sensitivity analysis.

The challenges of sparse data and complex ecological processes has been identified elsewhere in the literature. Sparse data is the norm in field studies, and renders parameter estimation for related models that seek to quantify detection and occupancy far more difficult that is generally acknowledged [18]. More work is needed to enable inference on population persistence to be based on more biologically plausible models. Approximate Bayesian Computation Techniques [33] offer promise as a method in this context.

Our lack of certainty shouldn't deter managers from applying quantitative methods such as the ones we have presented here and others in the literature to support inference on the success or otherwise of invasive species control programs. They should, however, treat the results cautiously, as such simple models may not necessarily provide good answers to potentially complex problems.

## Supporting Information

Code S1 **R code to run sampler for constant model.**
(R) Click here for additional data file.

Code S2 **R code to run sampler for non-constant model.**
(R) Click here for additional data file.
